# Nfat5 is involved in the hyperosmotic regulation of Tmem184b: a putative modulator of ibuprofen transport in renal MDCK I cells

**DOI:** 10.1002/2211-5463.12630

**Published:** 2019-05-07

**Authors:** Rune Nørgaard Rasmussen, Kenneth Vielsted Christensen, René Holm, Carsten Uhd Nielsen

**Affiliations:** ^1^ Department of Physics, Chemistry and Pharmacy University of Southern Denmark Odense M Denmark; ^2^ Neuropsychiatry Center for Therapeutic Innovation Servier Laboratories Croissy France; ^3^ Drug Product Development Janssens Research and Development Johnson & Johnson Beerse Belgium; ^4^ Department of Science and Environment Roskilde University Denmark

**Keywords:** hyperosmolality, ibuprofen carrier, NFAT5, siRNA, TMEM184b, transcriptomics

## Abstract

Nuclear factor of activated T cells 5 (NFAT5) is a transcription factor involved in the regulation of several genes involved in the response to extracellular hyperosmolality. Recently, the uptake of ibuprofen by an as yet unknown carrier was suggested in Madin‐Darby canine kidney (MDCK) I cells exposed to hyperosmolality. We therefore speculated that Nfat5 could be involved in the regulation of this ibuprofen carrier. Reverse transfection with siRNA against Nfat5 was used to knock down Nfat5 in MDCK I cells. The uptake of both radiolabelled taurine and ibuprofen was measured in MDCK I cells, first treated with siRNA against Nfat5 and afterwards cultivated with raffinose‐supplemented normal growth medium (500 mOsm) for 24 h. The siRNA transfection resulted in knockdown of Nfat5, and uptake of both taurine and ibuprofen was significantly decreased in transfected MDCK I cells. The decrease in ibuprofen uptake indicates that Nfat5 is involved in upregulation of the ibuprofen carrier. A transcriptome analysis of MDCK I cells treated with siRNA against Nfat5 revealed 989 genes upregulated by Nfat5 during hyperosmotic exposure. From these genes, the gene product transmembrane protein 184b was found to be regulated by Nfat5, and Tmem184b was the only potential gene product involved in the uptake of ibuprofen in MDCK I cells.

**Dataset:**

The RNA sequencing dataset is available from the NCBI Gene Expression 452 Omnibus (https://www.ncbi.nlm.nih.gov/geo/) with the accession number GSE122074.

**Abbreviations**
MDCK: Madin‐Darby canine kidney
NFAT5: nuclear factor of activated T cells 5
SLC: solute carrier
Tmem184b: transmembrane protein 184b
TonEBP: tonicity‐responsive binding‐protein

Nuclear factor of activated T cells 5 (NFAT5), also referred to as tonicity‐responsive binding‐protein (TonEBP), is a major transcriptional factor in the cellular response to hyperosmolality 1. Several genes are affected by NFAT5 during hyperosmotic exposure 2. *BGT1* (SLC6A12), *SMIT1* (SLC5A3), *TauT* (SLC6A6) and *aldose reductase* (AKR1B1) are particularly important genes regulated by NFAT5 3, 4, 5, 6. The proteins of the mentioned genes protect the cell during hyperosmolality by accumulating uncharged small organic osmolytes intracellularly. This process is vital, because the osmolytes can be accumulated in high concentrations with little effect on other cellular processes 7. Recently, an ibuprofen carrier was detected functionally in Madin‐Darby canine kidney (MDCK) I cells after exposure to hyperosmolality 8. The protein or proteins responsible for the cellular uptake of ibuprofen have not yet been identified. However, the upregulation of the underlying protein appears to be a result of increased transcription since the transcription inhibitor, actinomycin D, and the translation inhibitor, cycloheximide, both inhibit the upregulation of cellular ibuprofen accumulation 9.

A transcriptome analysis of MDCK I cells during hyperosmotic exposure has recently provided an overview of transporting proteins regulated by hyperosmolality 10. From this analysis, 25 candidate solute carriers (SLCs) were selected, and a siRNA knockdown approach was attempted without any effect on the hyperosmotic‐induced ibuprofen uptake in MDCK I cells (unpublished data). Therefore, further information about the regulatory signal upregulating ibuprofen uptake is needed in order to provide experimental tools to identify the underlying gene responsible for the ibuprofen transport in MDCK I cells. As several SLCs, such as SLC6A6, SLC5A3 and SLC6A12, are regulated by NFAT5 during hyperosmolality, we hypothesize that this transcription factor could also be involved in the upregulation of the ibuprofen carrier. Knowing the involvement of Nfat5 in the regulation of the ibuprofen transport will provide a possible new experimental approach to a new transcriptome analysis. This would potentially lower the number of candidates for the ibuprofen carrier. The aim of the present study is therefore to first investigate if a siRNA‐mediated knockdown of *Nfat5* in MDCK I cells affects the uptake of ibuprofen during hyperosmolality. And secondly, the aim is to use the obtained knowledge to perform a full transcriptome analysis aimed at identifying putative candidate genes coding for a carrier for ibuprofen. The findings of the study identified a set of 989 genes regulated by Nfat5 during hyperosmolality, and among these, transmembrane protein 184bB (Tmem184b) was investigated further. The upregulation of Tmem184b during hyperosmolality and the likely involvement of Nfat5 in the regulation improve the knowledge about the function and regulation of Tmem184b and suggest a role of the protein in ibuprofen transport.

## Materials and methods

### Materials

Dulbecco's modified Eagle's medium (DMEM)/F‐12, Tween 20, phosphate‐buffered saline solution, penicillin–streptomycin, siRNA constructs, d‐(+)‐raffinose pentahydrate, RIPA buffer, 2‐mercaptoethanol, 4‐(2‐hydroxyethyl)‐1‐piperazineethanesulphonic acid (HEPES), Triton X‐100, Trypsin–EDTA (×10) and l‐glutamine were all purchased from Sigma‐Aldrich (St. Louis, MO, USA). Hank's balanced saline solution (HBSS) (10×) and sodium bicarbonate (7.5%) were obtained from Gibco, Invitrogen (Paisley, UK). RS‐[^3^H(G)] ibuprofen ([^3^H]‐ibuprofen; 20 Ci·mmol^−1^) was from American Radiolabelled Chemicals, Inc. (St. Louis, MO, USA). [2,2‐^3^H(N)]‐taurine ([^3^H]‐taurine; 10.1 Ci·mmol^−1^) and Ultima Gold scintillation liquid were purchased from PerkinElmer (Boston, MA, USA). Cell culture plastic ware was obtained from Corning Life Science (Wilkes Barre, PA, USA). Water was obtained from a Milli‐Q water purification system (Merck Millipore, Darmstadt, Germany). FBS, Pierce™ BCA protein assay kit and Lipofectamine® RNAiMAX were from Thermo Fisher Scientific (Waltham, MA, USA). cOmplete™ ULTRA Tablets, Mini, *EASYpack* Protease Inhibitor Cocktail were purchased from Roche Diagnostics (Mannheim, Germany). Four to twenty percent Mini‐PROTEAN® TGX Stain‐Free™ Protein Gels, Trans‐Blot® Turbo™ Mini poly(vinylidene difluoride) (PVDF) Transfer packs, 10× Tris‐glycine‐SDS buffer, 10× Tris‐buffered saline, blotting‐grade blocker, Precision Protein™ StrepTactin‐HRP Conjugate, Precision plus protein™ WesternC™ protein standard, 4× Laemmli sample buffer and goat anti‐rabbit IgG (H + L)‐HRP Conjugate were obtained from Bio‐Rad (Hercules, CA, USA). Polyclonal rabbit anti‐Nfat5 antibody was from Novus biological.

### Cell cultivation

Madin‐Darby canine kidney I cells were obtained from European Collection of Authenticated Cell Cultures (ECACC, Porton Down, UK) and cultured using DMEM/F‐12 containing 1% penicillin–streptomycin, 2 mm l‐glutamine and 10% FBS (normal growth medium) in an atmosphere of 5% CO_2_‐ 95% O_2_ at 37 °C.

### siRNA knockdown

Madin‐Darby canine kidney I cells were transfected with siRNA by reverse transfection against *Nfat5* or *Tmem184b*. Briefly, siRNA (100 nm final concentration) and 1.5 μL Lipofectamine® RNAiMAX in a total volume of 100 μL serum‐free medium were prepared following the protocol of the manufacture and added to each well (24‐well plate, well area 1.9 cm^2^) and incubated at room temperature for approximately 20 min. Then, 400 μL cell suspension with a concentration of 427 500 MDCK I cells per mL (90 000 MDCK I cells per cm^2^) in normal growth medium was added. The cells were incubated at 37 °C 5% CO_2_ overnight. The next day, the medium containing siRNA was removed and exchange with normal growth medium overnight, whereafter raffinose‐supplemented (200 mOsm) normal growth medium (total osmolality of 500 mOsm) was added for 24 h. To control cells, Slc22a6‐siRNA (Neg‐siRNA) was used with cells treated with normal growth medium (300 mOsm) and raffinose‐supplemented normal growth medium (500 mOsm), as Slc22a6 is not expressed in MDCK I cells 10. After 24 h, the cellular uptake of either [^3^H]‐ibuprofen (50 nm, 1 μCi·mL^−1^) 1 or [^3^H]‐taurine (99 nm, 1 μCi·mL^−1^) was measured. When transfecting with siRNA against *Tmem184b*, the medium was not changed the day after transfection. Instead, the cells were treated with raffinose‐supplemented growth medium (500 mOsm) for 24 h resulting in a total cultivation time of 48 h. The sequences of the siRNA constructed used for siRNA transfection are given in Table 1.

**Table 1:** siRNA constructed used in siRNA knockdown

Accession number	Gene target	Nr	Direction	Sequence
XM_533258.5	Slc22a6 (Neg)	–	Sense (5′–3′)	CCAAUGGCUGGAUCUACGA
			Antisense (5′–3′)	UCGUAGAUCCAGCCAUUGG
XM_005620767.3	Nfat5	1	Sense (5′–3′)	GAATAATGGTACTCAGCAA
			Antisense (5′–3′)	UUGCUGAGUACCAUUAUUC
XM_005620767.3	Nfat5	2	Sense (5′–3′)	CUCACAUGAUGAGUGCAUU
			Antisense (5′–3′)	AAUGCACUCAUCAUGUGAG
XM_005620767.3	Nfat5	3	Sense (5′–3′)	CUUCUAAUCAGCUGCCCAA
			Antisense (5′–3′)	UUGGGCAGCUGAUUAGAAG
XM_005625888.3	Tmem184b	1	Sense (5′–3′)	GCAAGUACCGGGAUGGUGA
			Antisense (5′–3′)	UCACCAUCCCGGUACUUGC
XM_005625888.3	Tmem184b	2	Sense (5′–3′)	GCCUUCACCUACAAAGUCU
			Antisense (5′–3′)	AGACUUUGUAGGUGAAGGC
XM_005625888.3	Tmem184b	3	Sense (5′–3′)	CCAUCGAGUCCAGCUGUAU
			Antisense (5′–3′)	AUACAGCUGGACUCGAUGG

### Cellular uptake of ibuprofen and taurine

Before the uptake studies, the cells were equilibrated in prewarmed HBSS containing 10 mm HEPES and adjusted to pH 7.4 at 37 °C for 15 min. The cells were then incubated for 5 min at 37 °C with [^3^H]‐ibuprofen (50 nm, 1 μCi·mL^−1^) 1 or [^3^H]‐taurine (99 nm, 1 μCi·mL^−1^) in HBSS with 10 mm HEPES adjusted to pH 7.4 using 1 mm NaOH. After incubation, the uptake medium was removed and the cells were washed three times with ice‐cold HBSS. The cells were detached by addition of 200 μL 0.1% Triton X‐100 in Milli‐Q water. The cell suspension was transferred to pony vials, and 2 mL of Ultima Gold scintillation fluid was added followed by liquid scintillation counting using a TriCarp 4910 TR liquid scintillation counter from PerkinElmer.

### Western blotting

Total protein from MDCK I cells was isolated by washing the cells with ice‐cold PBS and afterwards treating with 10× RIPA buffer containing 1× protease inhibitor. The total protein content was measured using Pierce™ BCA protein assay following the protocol of the manufacture. Protein samples for western blotting were prepared by heating the protein to 95 °C for 5 min and adding 4× Laemmli sample buffer and 2‐mercaptoethanol to a final concentration of 1 mg·mL^−1^ protein, 1× Laemmli sample buffer and 1 : 39 2‐mercaptoethanol. The samples were loaded to a precast 4–20% polyacrylamide Stain‐Free™ gel and ran using 200 V in 1× Tris‐glycine‐SDS buffer. Precision plus protein™ WesternC™ protein standard was used as protein ladder. After running, the gel was activated in a ChemiDoc™ XRS+ imager (Bio‐Rad) and then blotted to a PVDF blot using trans‐blot® turbo™ blotter (Bio‐Rad). The blot was then treated with a 5% blocking solution (milk) in 1× TBST and then with primary antibody (1 : 1000) in a 5% blocking 1× TBST solution overnight at 4 °C on a rocker. The next day, secondary antibody (1 : 3000) was added in 5% milk in 1× TBST containing Precision Protein™ StrepTactin‐HRP Conjugate (1 : 5000). Following the treatment with secondary antibody, the HRP substrate was added to the blot and then visualized. The software Imager Lab™ (Bio‐Rad) was used to calculate the relative loaded protein and the relative band intensity.

### Real‐time PCR

Total RNA was isolated using Nucleospin® RNA/Protein (Macherey‐Nagel GmbH Co., Düren, Germany) following the protocol of the manufacture. cDNA was obtained by using High Capacity cDNA Reverse Transcription Kit, and Power SYBR® Green PCR Master Mix (Applied Biosystem, Foster City, CA, USA) was used for real‐time PCR, following the respective protocols. All reactions were run in duplicates. Primers (PentaBase, Odense, Denmark) were used at a concentration of 450 nm. The sequence of the primers used was as follows: *Nfat5* forward: ACCCAGAGACCCTGACAACT, *Nfat5* reverse: TGAAACTGGGTAGCCTGCTG, *Tmem184b* forward: GTGGAGATGTTCTTCGCAGC, *Tmem184b* reverse: GCCATACGTCGGCACTTG, *Gapdh* forward: GGAGGGCCTCATGACCACCGT and *Gapdh* reverse: CACATCTTCCCAGAGGGGCCGT.

### RNA sequencing

Madin‐Darby canine kidney I cells treated with siRNA and cultivated in isosmotic (300 mOsm) and hyperosmotic (raffinose; 500 mOsm) culture medium were used for RNA sequencing. RNA was isolated from the MDCK I cells using Nucleospin® RNA/Protein (Macherey‐Nagel GmbH Co.). The purity of the RNA was measured with a fragment analyser (AATI), following the protocol of the manufacturer. The library preparation was performed using NEBNext Ultra II RNA Library Prep Kit for Illumina (NEB), and the protocol of the manufacturer was followed. The sequencing was performed using a NovaSeq6000 (S2 flow cell) with paired end and a read length of 51. The quality of the sequence was evaluated using fastQC. The samples were mapped using Hisat 2.05 against the *Canis familiaris* 3.1 genome, received from Ensembl (http://www.ensembl.org/index.html). Each sample was counted using featureCounts with the 92 annotation of *Canis familiaris* 3.1. DESeq2 was used to perform the statistical analysis 11. All the samples were used to estimate the size factor and the dispersion. Genes, where the sum of counts was below five in all eight samples, were excluded. The contrast function in DESeq2 was then used to compare the individual conditions. An adjusted *P*‐value of 0.05 was used to determine whether a gene was differentially expressed in a condition. To adjust the *P*‐value for the false discovery rate, the Benjamini–Hochberg–Yekutieli method was used. The R‐package ‘pheatmap’ was used to create the heatmap of the RNA‐sequence analysis.

### Statistical analysis

All samples were performed in atleast biological triplicates, except the RNA sequencing that was performed in biological duplicates. Data are represented as mean ± SEM. Samples were compared by one‐way ANOVA, and Dunnet's multiple comparison test was used to determine the levels of statistical significance, where a *P*‐value < 0.05 was considered significant.

## Results

### siRNA knockdown of Nfat5 in MDCK I cells

**Figure 1:**
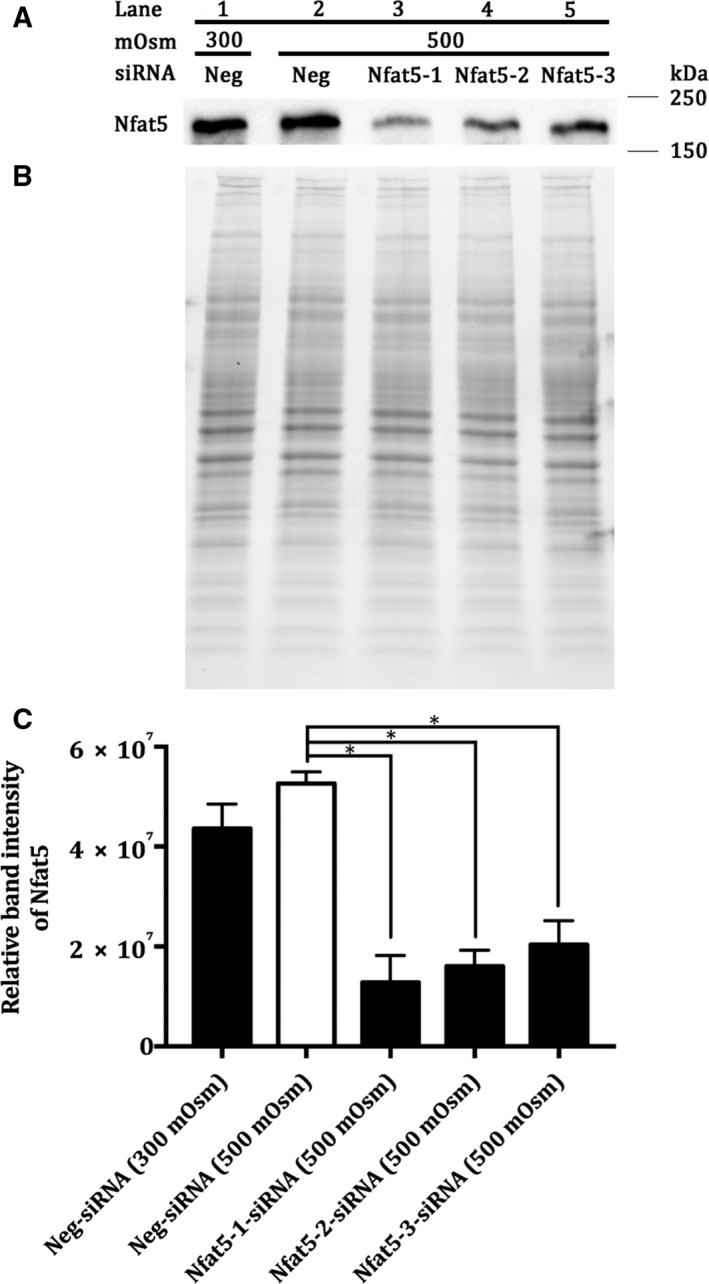
Protein expression of Nfat5 in Nfat5‐siRNA‐treated MDCK I cells cultivated under hyperosmotic conditions. (A) The protein expression of NFAT5, (B) total protein loading control and C: the relative band intensity of Nfat5 in MDCK I cells treated with lane 1: Neg‐siRNA (300 mOsm), lane 2: Neg‐siRNA (500 mOsm), lane 3: Nfat5‐1‐siRNA (500 mOsm), lane 4: Nfat5‐2‐siRNA (500 mOsm) and lane 5: Nfat5‐3‐siRNA (500 mOsm). The protein was separated on a 4–20% polyacrylamide Stain‐Free™ gel and immunoblotted using anti‐Nfat5 (1 : 1000). (A) The immunoblot represents an illustrative example of the results from three different cell passages, *n* = 3. (B) The total protein blot represents an illustrative example of the results from three different experiments, *n* = 3. (C) The band intensity was normalized to the total protein loaded using Imager Lab™ software. Each column represents the mean ± SEM of three different cell passages (*n* = 3). One‐way ANOVA followed by Dunnet's multiple comparison test was used to determine the levels of statistical significance (*P < 0.05).

**Figure 2:**
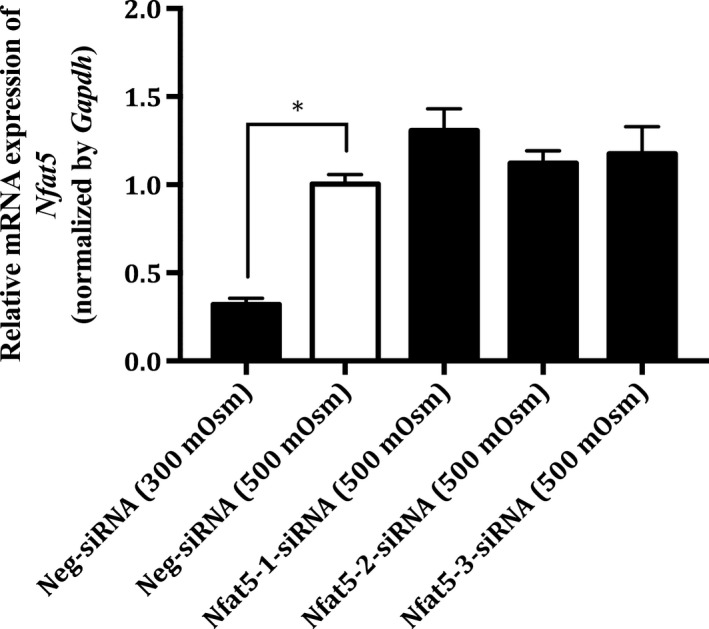
Relative mRNA expression of *Nfat5* in Nfat5‐siRNA‐treated MDCK I cells cultivated under hyperosmotic conditions. MDCK 1 cells were treated with (100 nm) Neg‐siRNA (300 mOsm), Neg‐siRNA (500 mOsm), Nfat5‐1‐siRNA (500 mOsm), Nfat5‐2‐siRNA (500 mOsm) and Nfat5‐3‐siRNA (500 mOsm) using raffinose‐supplemented growth medium as hyperosmotic condition. The MDCK I cells were exposed to hyperosmolality 48 h after the siRNA treatment and the hyperosmotic exposure lasted 24 h. The relative RNA level of *Nfat5* was measured by real‐time PCR and normalized by the expression of *Gapdh*. Each column represents the mean ± SEM of three different cell passages (*n* = 3). One‐way ANOVA followed by Dunnet's multiple comparison test was used to determine the levels of statistical significance (*P < 0.05).

The expression of *Nfat5* was knocked down in MDCK I cells using a siRNA approach. The effect of the knockdown at the protein level was investigated by western blotting (see Fig. 1A,B,C) and at the mRNA level by real‐time PCR (see Fig. 2).

As seen in Fig. 1A, the protein expression of Nfat5 was visually similar in the raffinose and the isosmotic treated cells. However, all three siRNA‐Nfat5 resulted in a decrease in the protein level of Nfat5, compared to the raffinose‐treated MDCK I cells. The amount of loaded protein in each well was determined using stain‐free precast gels and was similar in all wells (see Fig. 1B). The relative band intensity of Nfat5 (see Fig. 1C) showed that the Nfat5 expression was 3.3 times lower in Nfat5‐siRNA (500 mOsm)‐treated MDCK I cells compared to the Neg‐siRNA (500 mOsm). There was no significant change in the relative band intensity of Nfat5 Neg‐siRNA (500 mOsm) compared to Neg‐siRNA (300 mOsm).

There was no significant difference in the mRNA level of *Nfat5*, 72 h after transfection with each of the three *Nfat5* siRNAs. The level of *Nfat5* was 3.1 times higher in the hyperosmotic (500 mOsm)‐treated MDCK I cells, compared to the isosmotic (300 mOsm)‐treated cells, and the difference was statistically significant (*P* value = 0.002).

### siRNA‐mediated silencing of Nfat5 expression attenuates uptake of taurine and ibuprofen in hyperosmotic‐treated MDCK I cells

**Figure 3:**
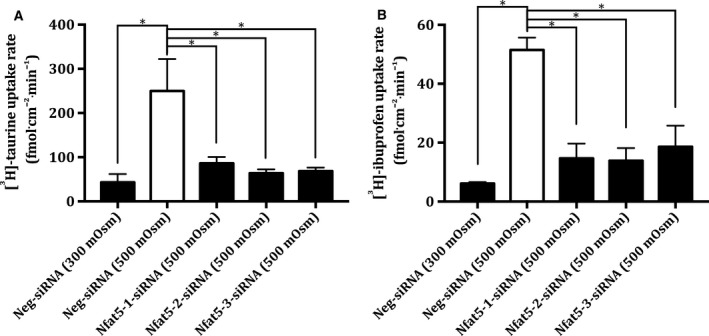
Cellular uptake of [^3^H]‐ibuprofen and [^3^H]‐taurine in Nfat5‐siRNA‐treated MDCK I cells cultivated under hyperosmotic conditions. Cellular uptake rate of 1 μCi·mL^−1^ [^3^H]‐taurine (99 nm) (A) and 1 μCi·mL^−1^ [^3^H]‐ibuprofen (B) (50 nm) in MDCK I cells treated with (100 nm) Neg‐siRNA (300 mOsm), Neg‐siRNA (500 mOsm), Nfat5‐1‐siRNA (500 mOsm), Nfat5‐2‐siRNA (500 mOsm) and Nfat5‐3‐siRNA (500 mOsm). The MDCK I cells were exposed to hyperosmolality 48 h after the siRNA treatment and the hyperosmotic exposure lasted 24 h. HBSS containing 10 mm HEPES adjusted to pH 7.4 with 1 mm NaOH was used as uptake buffer at 37°. The uptake was measured for 5 min. Each column represents the mean ± SEM of three to four different cell passages (*n* = 3–4). One‐way ANOVA followed by Dunnet's multiple comparison test was used to determine the levels of statistical significance (*P < 0.05).

The uptake of radiolabelled taurine and ibuprofen in MDCK I cells, treated with three different Nfat5‐siRNAs and raffinose, was investigated (see Fig. 3).

The results depicted in Fig. 3 show that the uptake of taurine and ibuprofen in raffinose‐ and Neg‐siRNA‐treated MDCK I cells was increased, compared to the cells cultivated under isosmotic conditions. Furthermore, the uptake of taurine and ibuprofen was decreased in MDCK I cells treated with any of the three Nfat5‐siRNAs (500 mOsm), compared to MDCK I cells treated with Neg‐siRNA (500 mOsm), respectively, thus confirming the involvement of Nfat5 in the hyperosmotic upregulation of both taurine and ibuprofen transport.

### Transcriptome analysis of Nfat5‐siRNA‐treated MDCK I cell under hyperosmotic conditions

**Figure 4:**
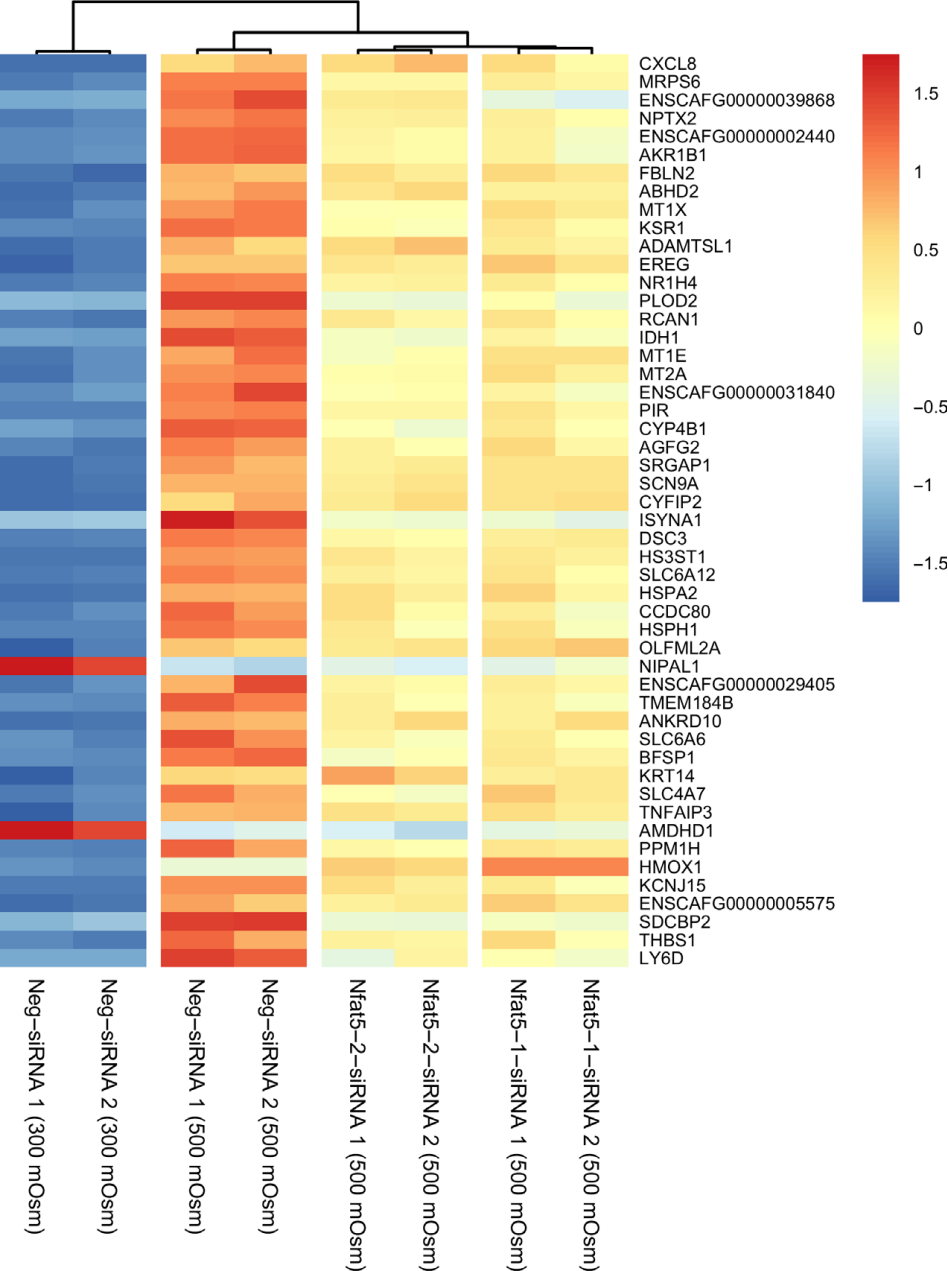
Heatmap of the top 50 genes affected by Nfat5‐siRNA and hyperosmotic treatment. The presented genes are the top 50 genes significantly (*P*‐adjusted < 0.05) upregulated by hyperosmolality (500 mOsm) compared to the isosmotic (300 mOsm) and simultaneously significantly decreased (*P*‐adjusted < 0.05) by both Nfat5‐siRNA treatments (500 mOsm) compared to the hyperosmotic condition (500 mOsm). The colour of each cell represents the relative expression of the gene in the specific condition compared to the average expression of all conditions. The genes are ordered according to the *P*‐adjust value of upregulated genes from the hyperosmotic condition (500 mOsm) compared to the isosmotic condition (300 mOsm). The statistical analysis was performed with DESeq2, and the false discovery rate was controlled by the Benjamini–Hochberg–Yekutieli method with a significance level of 0.05. *n* = 2 passages (each represented in the figure). The heatmap was generated using the R‐package ‘Pheatmap’.

A transcriptome analysis of Nfat5‐siRNA‐treated MDCK I cells under hyperosmotic conditions was performed to identify possible candidates for the responsible gene of the ibuprofen carrier. A total of 989 genes were significantly (*P*‐adjusted < 0.05) upregulated by hyperosmolality (500 mOSm) compared to the isosmotic condition (300 mOsm) and simultaneously significantly decreased (*P*‐adjusted < 0.05) by both Nfat5‐siRNA (construct 1 and 2) treatments (500 mOsm) compared to the hyperosmotic condition (500 mOsm). Of the 989 genes, the top 50 regulated genes are shown as a heatmap in Fig. 4. A complete list of the 989 genes is found in Table S1.

A hierarchical cluster analysis of the transcriptomes (data not shown) showed a similar cluster tree as the one in the heatmap (see Fig. 4).

Notably, the SLCs *Slc6a6*,* Slc6a12* and *Slc5a3* as well as the enzyme *Akr1b1* had a significant (*P*‐adjusted < 0.05) involvement of Nfat5 in the upregulation during hyperosmolality. These findings are important because Nfat5 regulates Slc6a6, Slc6a12, Slc5a3 and Akr1b1 during hyperosmolality. mRNA expression of *Nfat5* was significantly (*P*‐adjusted < 0.05) upregulated 3.5 times by hyperosmolality (500 mOsm) compared to the isosmotic condition (300 mOsm), but the Nfat5‐siRNA showed no effect on the Nfat5 mRNA level compared to the hyperosmotic (500 mOsm) condition.

The 989 potentially Nfat5 regulated genes were compared to the Pfam protein families database 12, and this revealed that Tmem184b is in the Pfam protein family Solute_trans_a (PF03619). The Solute_trans_a family is the family of the organic solute transporter subunit alpha (Ost_alpha_).

### Nfat5 is involved in the regulation of Tmem184b during hyperosmotic exposure

**Figure 5:**
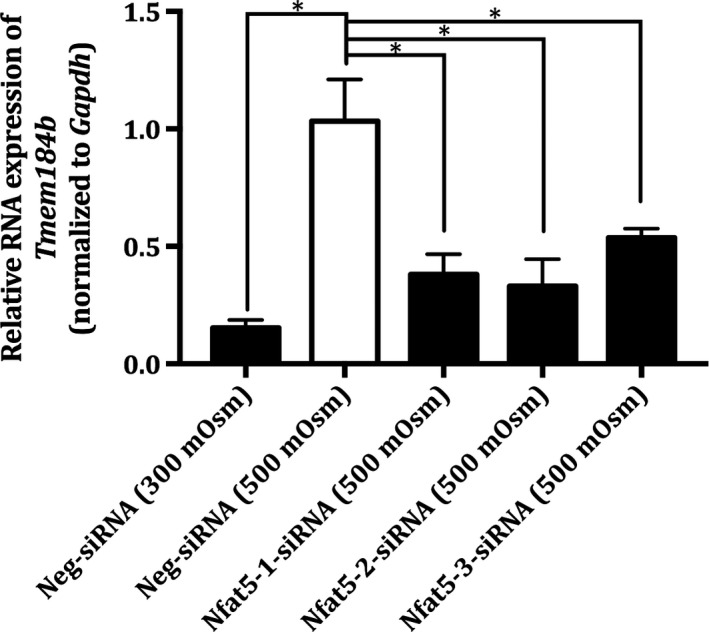
Relative mRNA expression of *Tmem184b* in Nfat5‐siRNA‐treated MDCK I cells cultivated under hyperosmotic conditions. MDCK 1 cells were treated with (100 nm) Neg‐siRNA (300 mOsm), Neg‐siRNA (500 mOsm), Nfat5‐1‐siRNA (500 mOsm), Nfat5‐2‐siRNA (500 mOsm) and Nfat5‐3‐siRNA (500 mOsm). The MDCK I cells were exposed to hyperosmolality 48 h after the siRNA treatment and the hyperosmotic exposure lasted 24 h. The relative RNA level of *Tmem184b* was measured by real‐time PCR and normalized by the expression of *Gapdh*. Each column represents the mean ± SEM of three different cell passages (*n* = 3). One‐way ANOVA followed by Dunnet's multiple comparison test was used to determine the levels of statistical significance (*P < 0.05).

A validation of the association between Nfat5 and *Tmem184b* was performed by real‐time PCR (see Fig. 5).

The real‐time PCR analysis of the *Tmem184b* expression showed that there was a significant 6.7 times upregulation of *Tmem184b*, when MDCK I cells were exposed to hyperosmolality (500 mOsm) compared to the isosmotic condition (300 mOsm). Furthermore, the transfection of MDCK I cells with all three Nfat5‐siRNAs resulted in a 2.3 times lower *Tmem184b* expression compared to the hyperosmotic‐treated MDCK I cells treated with Neg‐siRNA.

### Tmem184b could be partly responsible for the ibuprofen uptake in hyperosmotic‐treated MDCK I cells

**Figure 6:**
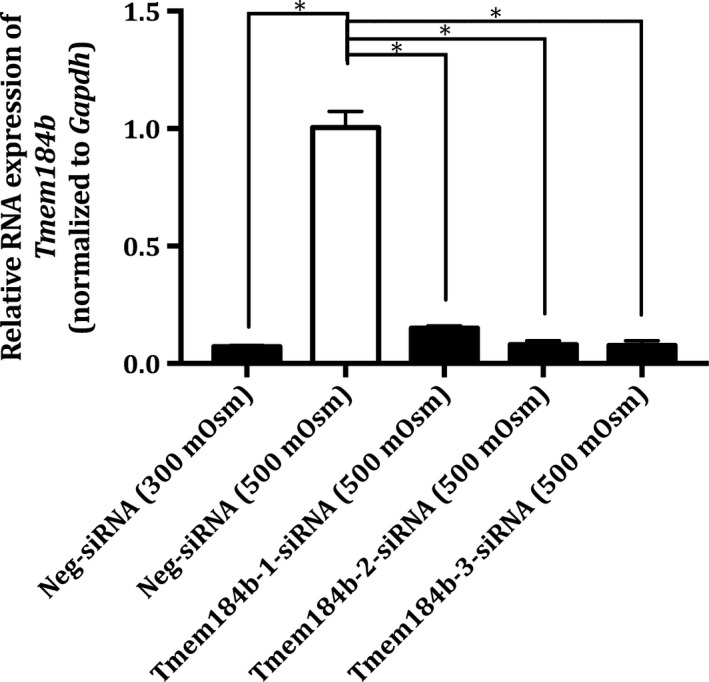
Relative mRNA expression of *Tmem184b* in Tmem184b‐siRNA‐treated MDCK I cells cultivated under hyperosmotic conditions. MDCK 1 cells were treated with (100 nm) Neg‐siRNA (300 mOsm), Neg‐siRNA (500 mOsm), Tmem184b‐1‐siRNA (500 mOsm), Tmem184b‐2‐siRNA (500 mOsm) and Tmem184b‐3‐siRNA (500 mOsm). The MDCK I cells were exposed to hyperosmolality 24 h after the siRNA treatment and the hyperosmotic exposure lasted 24 h. The relative RNA level of *Tmem184b* was measured by real‐time PCR and normalized by the expression of *Gapdh*. Each column represents the mean ± SEM of three different cell passages (*n* = 3). One‐way ANOVA followed by Dunnet's multiple comparison test was used to determine the levels of statistical significance (*P < 0.05).

The discovery that Tmem184b belongs to the Solute_trans_a Pfam protein family led to the hypothesis that Tmem184b can be involved in the hyperosmotic‐induced ibuprofen uptake either as a regulatory factor or as the actual protein responsible for ibuprofen transport. The mRNA level of *Tmem184b* during the hyperosmotic exposure was knocked down using siRNA against *Tmem184b* (see Fig. 6).

The real‐time PCR analysis in Fig. 6 shows that the knockdown of *Tmem184b* resulted in an average of 10 times lower mRNA expression of *Tmem184b* compared to MDCK I cells treated with Neg‐siRNA (500 mOsm). Furthermore, the level of *Tmem184b* was 14 times higher in the hyperosmotic‐treated MDCK I cells compared to the isosmotic treated.

**Figure 7:**
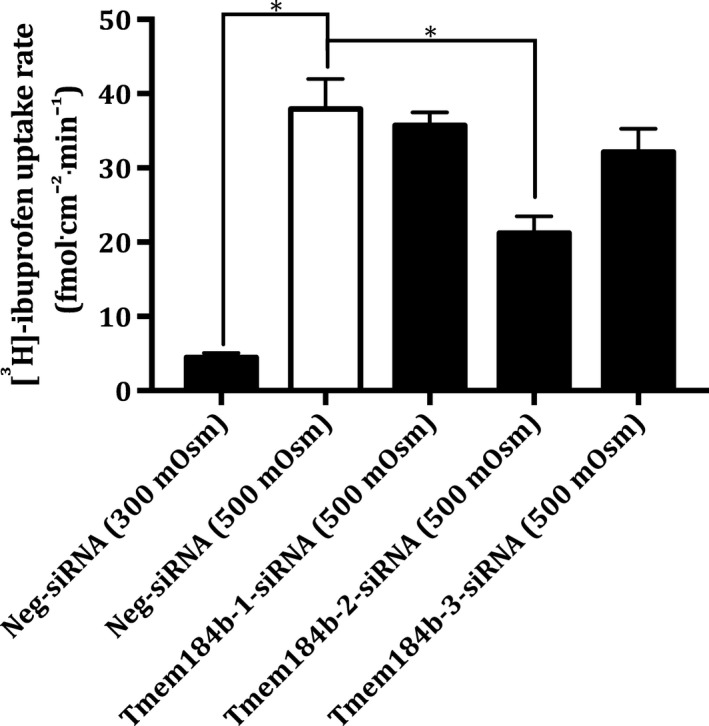
Cellular uptake of [^3^H]‐ibuprofen in Tmem184b‐siRNA‐transfected MDCK I cells cultivated under hyperosmotic conditions. Cellular uptake rate of 1 μCi·mL^−1^ [^3^H]‐ibuprofen (50 nm) in MDCK I cells treated with (100 nm) Neg‐siRNA (300 mOsm), Neg‐siRNA (500 mOsm), Tmem184b‐1‐siRNA (500mOsm), Tmem184b‐2‐siRNA (500 mOsm) and Tmem184b‐3‐siRNA (500 mOsm). The MDCK I cells were exposed to hyperosmolality 24 h after the siRNA treatment and the hyperosmotic exposure lasted 24 h. HBSS containing 10 mm HEPES adjusted to pH 7.4 with 1 mm NaOH was used as uptake buffer at 37°. The uptake was measured for 5 min. Each column represents the mean ± SEM of three different cell passages (*n* = 3). One‐way ANOVA followed by Dunnet's multiple comparison test was used to determine the levels of statistical significance (*P < 0.05).

The ibuprofen uptake in Tmem184b‐siRNA‐transfected MDCK I cells was measured after the exposure of hyperosmolality for 24 h (see Fig. 7).

The ibuprofen uptake was 8.4 times higher in the hyperosmotic (500 mOsm)‐treated MDCK I cells compared to the isosmotic (300 mOsm)‐treated MDCK I cells, when they were transfected with Neg‐siRNA, which was significantly different. Only Tmem184b‐2‐siRNA resulted in a significant decrease of the ibuprofen uptake in hyperosmotic (500 mOsm)‐treated MDCK I cells. The two other Tmem184b‐siRNA constructs had no significant effect on the ibuprofen uptake.

## Discussion

### Nfat5 is involved in the upregulation of an ibuprofen carrier in hyperosmotic‐treated MDCK I cells

The NFAT family consists of five proteins, that is NFAT1 (NFATc2 or NFATp), NFAT2 (NFATc1 or NFATc), NFAT3 (NFATc4), NFAT4 (NFATc3 or NFATx) and NFAT5 (TonEBP or OREBP) 13, whereof NFAT5 is important during hyperosmolality. As a response to hyperosmolality, mammalian cells upregulate the SLCs Slc6a6, Slc5a3 and Slc6a12 14, 15, 16. These carriers are, in part, responsible for the survival of the cell during states of hyperosmolality by accumulating uncharged small organic osmolytes. Nfat5 is the transcription factor involved in the osmotic regulation of Slc6a6, Slc5a3 and Slc6a12 3, 5, 6. During hyperosmolality, the level of NFAT5 in the nucleus increases. This is caused by an upregulation of NFAT5 and a translocation of NFAT5 from the cytoplasm to the nucleus 17.

In the present study, we investigated whether NFAT5 was involved in the regulation of an ibuprofen carrier functionally upregulated in MDCK I cells during hyperosmolality. A siRNA knockdown approach was used. A previous study found the protein level of NFAT 5 decreased 2 and 3 days after transfection with siRNA against NFAT5, whereas the level of NFAT5 was normal after 7 days 3. Similarly, we found that the protein level of Nfat5 decreased 3 days after transfection; however, the mRNA level was not significantly affected. The contradiction of the decreased protein level and unaffected mRNA may be because the effect of the siRNA knockdown is starting to lose effectiveness, which would first be seen at the mRNA level and later at the protein level. The known Nfat5 regulated SLCs *Slc6a6*,* Slc5a3* and *Slc6a12* as well as the enzyme *Akr1b1* were all found downregulated in Nfat5‐siRNA‐transfected cells in the transcriptome analysis. These results combined with the decreased function of Slc6a6, seen by a decrease in the taurine uptake, show that the Nfat5‐siRNA‐transfection provided a system with loss of function of Nfat5. The decreased ibuprofen uptake of siRNA‐Nfat5‐transfected MDCK I cells, therefore indicated that Nfat5 was involved in the upregulation of the responsible gene after exposure to hyperosmotic conditions. It could further indicate that the ibuprofen carrier was somewhat involved in the cellular response to hyperosmolality in MDCK I cells due to the role of Nfat5 during hyperosmolality. However, to confirm this further studies are needed.

### Tmem184b could be involved in the increased ibuprofen uptake in hyperosmotic‐treated MDCK I cells

Transporting proteins detected functionally, but with no knowledge of the underlying gene responsible, have traditionally been identified using expression cloning 14, 16, 18, 19, 20. We tried a similar approach to identify the ibuprofen carrier, with enrichment of mRNA by fractionation on a sucrose gradient and then expressed in *Xenopus laevis oocytes* (data not shown). However, the result showed no significant difference in the ibuprofen uptake in *Xenopus laevis oocytes* injected with the fractions of mRNA from hyperosmotic‐treated MDCK I cells. One complicating factor for analysing data on ibuprofen uptake in *Xenopus laevis oocytes* is that ibuprofen had a high passive transport in this system. Therefore, it was difficult to identify a low‐capacity carrier such as the ibuprofen carrier 8. A different approach was therefore needed to identify possible candidates for the ibuprofen carrier. The ibuprofen carrier is most likely upregulated transcriptionally 9. A transcriptome analysis of genes upregulated in hyperosmotic‐treated MDCK I cells should include the underlying transcript responsible for the ibuprofen uptake. Previously, we have identified the SLCs regulated by hyperosmolality in MDCK I cells 10. A siRNA approach to knock down each of the identified SLCs did not result in a potential candidate for the ibuprofen carrier (data not shown). It was therefore expected that the responsible gene was not currently annotated as a SLC. With the additional knowledge of the involvement of Nfat5 in the regulation of the ibuprofen carrier, we performed a transcriptome analysis to identify genes regulated by Nfat5 during hyperosmolality in MDCK I cells. The set of Nfat5 regulated genes was run against the Pfam protein family database to identify whether they belonged to a SLC family. The analysis revealed that, of the genes listed, Tmem184b is part of the Solute_trans_a family (PF03619). Solute_trans_a is the family of organic solute transporter alpha (SLC51A). SLC51A is the alpha subunit of the Ost_alpha_‐Ost_beta_ heterodimer carrier 20. The Ost_alpha_‐Ost_beta_ is responsible for the basolateral bile acid and steroid transport in the intestinal, renal and biliary epithelia 21. The physiological role of Tmem184b is still unknown, but a few studies have investigated Tmem184b 22, 23. TMEM184b is expressed in the nervous system, and it was suggested present in the plasma membrane and necessary for the maintenance of the peripheral nerve terminal and its morphology *in vivo* 22. Another study found TMEM184b upregulated in cancer, and that it is important for the migration and invasion of cancer *in vitro* 23. The family resembles between Tmem184b and Slc51a could reveal that Tmem184b might, in part, be involved for the ibuprofen uptake in MDCK I cells exposed to hyperosmolality. Further, resemblance between Ost_alpha_ and the ibuprofen carrier is that they are both anion carriers and sodium independent 8, 24. However, the uptake of anionic ibuprofen in hyperosmotic‐treated MDCK I cells is saturable both at the apical and at basolateral membrane, whereas Ost_alpha_‐Ost_beta_ is a basolateral carrier 9. As only one siRNA construct against *Tmem184b* had an effect on the uptake of ibuprofen, but all three knocked down the mRNA level of *Tmem184b*, it was not possible to unequivocally determine whether Tmem184b is partly responsible for the uptake of ibuprofen. The absent of attenuated ibuprofen uptake after treatment with two of the siRNA constructs against *Tmem184b* could possibly be explained by a lack of change in phenotype despite a substantial attenuation of the mRNA level of Tmem184b. *Tmem184b* was also found upregulated 9.8 and 7.0 times in a 12‐replica transcriptome analysis of hyperosmotic‐treated MDCK I cell caused by both raffinose (500 mOsm) and NaCl (500 mOsm), respectively, but no effect was seen by urea confirming the transcription osmotic regulation of Tmem184b 10.

## Conclusions

The present study showed that Nfat5 is involved in the regulation of the functional upregulation of ibuprofen transport in MDCK I cells during hyperosmotic exposure. By transcriptome analysis, a set of genes were identified, which were regulated by Nfat5 during hyperosmolality among these also Tmem184b. The upregulation of Tmem184b during hyperosmolality and the likely involvement of Nfat5 in the regulation improve the knowledge regarding Tmem184b about its function and regulation. The present study further suggested that Tmem184b is partly involved in the uptake of ibuprofen in hyperosmotic‐treated MDCK I cells.

## Conflict of interest

The authors declare no conflict of interest.

## Author contributions

RNR contributed to the design of the study, data collection, data analysis, and data interpretation, and the drafting of the manuscript. CUN contributed to the design of experiment, data interpretation and drafting of the manuscript. KVC contributed to the design of the experiment, data interpretation and drafting of the manuscript. RH contributed to the design of the experiment, data interpretation and drafting of the manuscript. All authors read and approved the final manuscript.

## Supporting information


**Table S1.** List of genes regulated by Nfat5 in hyperosmotic‐treated MDCK I cells during hyperosmolality. The list includes the genes significantly (*P*‐adjusted < 0.05) upregulated by hyperosmolality (500 mOsm) compared to the isosmotic (300 mOsm) medium and simultaneously significantly decreased (*P*‐adjusted < 0.05) by both Nfat5‐siRNA treatments (500 mOsm) compared to the hyperosmotic condition (500 mOsm). The statistical analysis was performed with DESeq2, and the false discovery rate was controlled by the Benjamini–Hochberg–Yekutieli method with a significance level of 0.05. *n* = 2 passage.
